# A Rare Case of a Solitary Fibrous Tumor of the Spermatic Cord

**DOI:** 10.1155/2021/9956305

**Published:** 2021-05-22

**Authors:** Moyosore Awobajo, Stefanie Hettwer, Sarah Hackman

**Affiliations:** University of Texas Health Science Center at San Antonio, 7703 Floyd Curl Drive, MC 7750, San Antonio, TX 78229, USA

## Abstract

Solitary fibrous tumors (SFTs) are rare mesenchymal tumors, originally identified in the pleura. Even though they have subsequently been described in several extrapleural sites, the incidence of SFTs in the spermatic cord is particularly rare. Here, we report a case of a 27-year-old male that presented with a 3-year history of left scrotal swelling. Computed tomography (CT) and ultrasound demonstrated multiple solid, hypoechoic well-circumscribed masses that were separate from the testis. Surgical excision of the mass led to pathologic diagnosis of a solitary fibrous tumor involving the spermatic cord. Solitary fibrous tumors, although rare, are an important differential diagnosis for urogenital tumors.

## 1. Introduction

Solitary fibrous tumors (SFTs) are rare fibroblastic mesenchymal neoplasms, first described in 1931 by Klemperer and Rabin [1]. It was first discovered in the pleura but has since been reported in various extrathoracic sites, accounting for 50-70% of all SFTs [2–4]. There are few reported cases of urogenital SFTs in the English medical literature, particularly those involving the spermatic cord. Here, we report a case of a 27-year-old male with SFT of the spermatic cord and its pathological characteristics.

## 2. Case Report

A 27-year-old man presented with a 3-year history of an enlarging left scrotal swelling and scrotal pain. He denied a history of cryptorchidism, hematuria, and epididymitis and had no prior history of urologic intervention.

**Figure 1:**
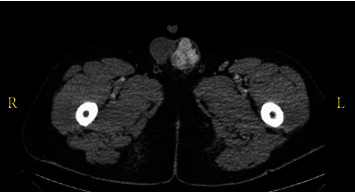
CT scan of the abdomen and pelvis revealed a large lobulated mass in the left scrotum.

CT of the abdomen and pelvis revealed a large lobulated hypervascular left scrotal lesion measuring 3.9 × 3.7 cm with associated left varicocele (Figure 1). Ultrasonography (US) of the scrotum demonstrated either multiple solid, hypoechoic well-circumscribed masses that were separate from the left testis or a single lobulated mass. On doppler ultrasound, normal vascularity was demonstrated. Because of the concern for a germ cell tumor, beta-HCG, alpha-fetoprotein (AFP), and lactate dehydrogenase (LDH) tumor markers performed and were negative. Upon inguinal exploration, the testis was atrophic, and the mass appeared to be separate from the testis and arising from the spermatic cord. The mass was surgically excised with preservation of the testis and submitted for pathologic examination.

**Figure 2:**
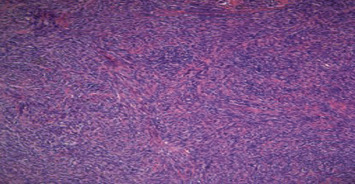
Hematoxylin and eosin stain demonstrating a patternless spindle cell neoplasm (×10 magnification).

**Figure 3:**
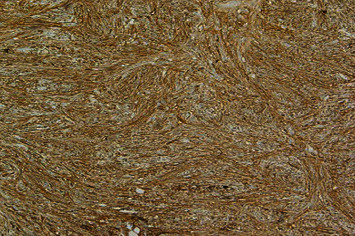
CD34 IHC stain demonstrating strong diffuse positivity (×10 magnification).

**Figure 4:**
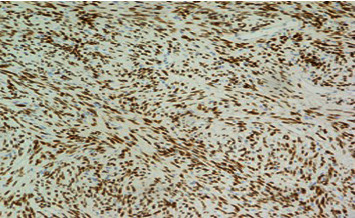
STAT6 IHC stain demonstrating strong diffuse positivity (×20 magnification).

On gross examination, the specimen received was a firm, nodular, tan-white soft tissue fragment measuring 4.8 cm with no areas of hemorrhage or necrosis. Histologic examination revealed a spindled cell neoplasm (Figure 2) with moderate cytologic atypia and a focal area of increased cellularity with up to 4 mitoses per high-power field. Tumor cells were strongly positive for CD34 (Figure 3), BCL-2, and STAT6 (Figure 4). Focal and/or patchy staining was seen with ER, PR, SMA, and CD117. EMA, DOG-1, Desmin, and EBER in situ hybridization (ISH) were negative in the neoplastic cells. Based on the histopathologic and immunohistochemical findings, the diagnosis of solitary fibrous tumor (SFT) of the left spermatic cord was made. According to the risk model outlined in the World Health Organization (WHO), this tumor is classified as low risk. However, areas of the tumor abutted the resection margin.

Unfortunately, at five months post-follow-up, the patient refused further medical services against medical advice and was lost to follow-up.

## 3. Discussion

SFTs are mesenchymal tumors, first described in 1931 by Klemperer and Rabin and further classified by Briselli in 1989 [1]. SFTs are rare and only account for less than 2% of all soft-tissue tumors. SFTs are most often seen in the fifth to seventh decades of life but can occur at any age [5]. First described in the pleura, SFTs were historically termed as solitary fibrous mesothelioma, submesothelial fibroma, or pleural fibroma. According to the revised WHO's *Classification of Tumors*, SFTs are currently described as soft tissue tumors of pluripotent fibroblastic origin that can arise in any part of the body [6].

While extrathoracic sites such as abdomen [7], pelvis, meninges [8], head and neck [9], and soft tissue of extremities [10] have since been described as common locations for this neoplasm, there have been very few reported cases of SFTs arising in the spermatic cord [11].

SFTs can be detected through physical examination or imaging, but histologic analysis is required for a diagnosis [1]. SFTs that develop in extrathoracic sites possess similar histological characteristics as pleural SFTs [12]. Macroscopically, these tumors are well-circumscribed encapsulated firm masses with a whorled fibrous appearance. Foci of cystic degeneration, hemorrhage, necrosis, and dystrophic calcification are possible. Histologically, these tumors present as alternating hypercellular and hypocellular areas of spindled cells interspersed amongst thick bands of collagen fibers and distinct networks of anastomosing vessels [13].

SFTs develop from submesothelial spindle stromal cells and are mostly considered to be benign; however, a retrospective study including SFTs from all anatomic location has shown a number of SFTs to be malignant with substantial rates of local recurrence, metastasis, and mortality. The factors associated with increased risk of local recurrence and metastasis include positive resection margins, tumor size greater than 10 cm, nuclear pleomorphism, increased cellularity, and an increased mitotic rate (>4 mitoses per 10 high-power fields) [2]. Long-term follow-up is advised in all SFT cases due to the possibility of a late recurrence and/or malignant potential.

Histopathologic diagnosis of SFTs that develop in paratesticular tissue could be diagnostically challenging due to their similarity with other spindle cell fibroblastic tumors such as fibrosarcoma, angiomyolipoma, leiomyoma, nerve sheath tumor, and adenomatoid tumor. Immunohistochemical (IHC) analysis therefore plays an important role in diagnosis of SFTs, especially those that arise from extrathoracic sites. CD34 and Bcl-2 are highly sensitive for SFTs, with positivity seen in >80% of cases [14, 15]. CD99 positivity is also a hallmark for SFT diagnosis [16]. CD34 is a marker for benign endothelium and vascular tumors while Bcl-2 is a marker of terminal differentiation. These IHC markers are therefore not specific for SFTs and may also be present in other mesenchymal tumors. NAB2-STAT6 fusion gene expression was first described in 2013 as a unique molecular marker present in most SFTs [17]. More recent reports have demonstrated STAT6 IHC nuclear positivity as a useful diagnostic tool to differentiate SFTs from other tumors with similar histologic characteristics [18].

In malignant SFTs, S-100, cytokeratin, vimentin, and p53 IHC markers have reportedly been positive [5].

The conventional treatment modality for localized SFT is surgical resection with negative margins. Reported 5-year survival rate postresection of both pleural and extrathoracic SFTs range from 73 to 100 percent [5]. Cases of locally aggressive/recurrent and malignant SFTs may benefit from chemotherapy or radiation, despite the lack of well-established treatment outcomes when used in treatment of SFTs [16].

Our patient was considered to have a benign/low risk SFT due to its small size (<10 cm) and mostly benign histologic features and immunohistochemical staining pattern. However, positive resection margins are concerning for future local recurrence. During the 5-month follow-up period, the patient remained healthy and exhibited no tumor recurrence.

## 4. Conclusion

Solitary fibrous tumors are rare neoplasms that occur at all anatomic sites. However, very few reports have been published of SFTs of paratesticular origin, particularly those arising from the spermatic cord. Therefore, long-term follow-up data is not readily available upon which to base evidence for treatment validity. However, based on the known disease course and favorable prognosis of SFTs at other anatomic locations, spermatic cord SFTs with low proliferative index are expected to have a low propensity for local recurrence and metastasis. Due to the rarity of SFTs of the spermatic cord, reporting of clinical presentations, diagnosis, management, and long-term monitoring of these patients with physical examinations and serial scrotal ultrasounds are pertinent to evaluate pattern of disease progression and health outcomes at this anatomic site.
